# Nurturing scholarship: Reflections on the inaugural *South African Family Practice* Editorial Fellowship

**DOI:** 10.4102/safp.v68i1.6250

**Published:** 2026-03-02

**Authors:** Simon M. Marcus, Mmaphefo M. Maluleka

**Affiliations:** 1 Department of Family Medicine and Primary Care, Faculty of Health Sciences, University of the Witwatersrand, Johannesburg, South Africa; 2 Department of Family Medicine and Primary Health Care, Faculty of Medicine, Sefako Makgato Health Sciences University, Pretoria, South Africa

## Background

The importance of clinician-scholars is increasingly recognised globally because of their unique ability to bridge the gap between clinical practice and academic research.^1,2^ With the exponential increase in medical research, translating evidence into practical action has become essential to ensure effective, evidence-based care that improves patient and community health.^3^ This is particularly important within family medicine and primary care, where the majority of healthcare occurs.^4^

One of the key steps in producing and disseminating high-quality research is the scientific publishing process, including editorial and peer review. To help nurture early-career clinician-scholars in family medicine and primary care, the *South African Family Practice* journal launched its Editorial Fellowship in 2024.^5^

The Fellowship is available to all currently enrolled South African Family Medicine registrars with a demonstrated interest in primary care research and has four main objectives:

- *To develop editorial skills* through manuscript selection, peer review analysis and engagement in publication ethics
- *To strengthen critical appraisal and writing* through the production of high-quality editorials and the assessment of peer reviews
- *To enhance leadership skills* by actively contributing to journal strategy, outreach and innovation
- *To foster professional collaboration* by linking fellows with national and international family medicine researchers and editors

## Reflection

As our Fellowship draws to a close, we reflect on our experiences and the value this opportunity added to our knowledge and professional growth.

We had a unique opportunity to shape the Fellowship as inaugural fellows, working collaboratively with the editorial board to create its outline and objectives. Through this process, we were able to identify gaps in our training programmes that this Fellowship is uniquely placed to fill.

Each fellow was paired with a senior editor, who is responsible for a particular section of the journal. With their guidance, we managed submissions to their section from start to finish, including initial assessment of the submissions, assigning and following up with peer reviewers, reviewing the assessments, making final decisions and communicating with authors and journal administration. This helped us to develop a deeper understanding of the review process and the critical elements that ensure research credibility and integrity. We were able to discuss our analysis of peer reviews extensively with our senior editor, who provided expert guidance on how to approach reviewing articles and reviewing the review. Both activities were transformative in helping us hone our skills in critical research appraisal.

We joined bimonthly meetings of the editorial team, where all submissions were discussed. These vibrant conversations highlighted the collaborative nature of the publication process and offered valuable insights into addressing ethical issues such as ‘salami slicing’ or the responsible use of artificial intelligence (AI). They also provided the platform for reflection on the journal itself, which we embraced wholeheartedly. Through this engagement we were able to make meaningful contributions to discussions on enhancing the journal’s reach and relevance, particularly for our fellow registrars and students, and propose strategies to improve the translation of research and increase its impact among policymakers and the public.

Each fellow had the opportunity to pursue a special interest project, with one focusing on the journal’s creative design and cover art and the other on developing a podcast for the journal. We worked on these passion projects, which contributed to the journal’s growth, boosted our confidence, and helped us strengthen our leadership skills. Co-authoring an editorial on the journal’s commitment to primary care,^5^ published earlier this year, also sharpened our writing abilities and reinforced our sense of belonging. This momentum led one of the fellows to take the lead on an upcoming continuing professional development (CPD) article, which is set to be published next year.

One of the highlights of the Fellowship was our participation at the *27th SA Academy of Family Physicians* conference, where we helped celebrate the journal’s 45th anniversary. One of the fellows had the privilege of joining other editorial team members in the *South African Family Practice* panel discussion, where they highlighted the journal’s plans to help shape the future of family medicine and primary healthcare research.

## Conclusion

The inaugural *South African Family Practice* journal’s Editorial Fellowship has been a transformative and career-shaping experience. It provided structured mentorship, deepened our understanding of editorial processes, and enhanced our scholarly communication and leadership skills. None of these would have been possible without the welcoming and collaborative spirit of the editorial team and editor-in-chief, who fostered a genuine sense of belonging and reinforced our commitment to advancing research.

As the journal marks its 45th anniversary, this Fellowship is a powerful example of how targeted capacity-building initiatives can help cultivate the next generation of family medicine clinician-scholars and expand the impact of South African primary care scholarship.

The call for 2026 fellows will open soon. If you are a current family medicine registrar with a passion for research and advocacy for our discipline, this opportunity is for you.
